# Effect of Perioperative Blood Transfusion on Complications Following Emergency Non-trauma Laparotomy in Mulago Hospital: A Prospective Cohort Study

**DOI:** 10.7759/cureus.65759

**Published:** 2024-07-30

**Authors:** Flavius E Egbe, Richard N Iranya, Christian A Dimala, Ronald Mbiine, Michael Okello, Paul K Okeny

**Affiliations:** 1 Surgery, Makerere University College of Health Sciences, Kampala, UGA; 2 Surgery, Moyo General Hospital, Moyo, UGA; 3 Cardiovascular Medicine, The University of Texas Medical Branch, Galveston, USA; 4 Anatomy, Makerere University College of Health Sciences, Kampala, UGA

**Keywords:** mulago hospital, emergency non-trauma laparotomy, mortality, surgical site infection, perioperative blood transfusion

## Abstract

Background

Although blood transfusion may be required during emergency non-trauma laparotomy, several retrospective cohort studies have identified blood transfusion as a significant predictor of postoperative infections and mortality. However, no study has explored such an association in a resource-limited setting. This study aims to determine the effect of perioperative blood transfusion on the 30-day risk of surgical site infections (SSIs) and mortality among patients undergoing emergency non-trauma laparotomy in a large urban tertiary hospital in a resource-limited setting.

Methodology

In this prospective, single-center, cohort study, we recruited 160 consecutive adult patients admitted to the general surgery wards 48 hours after emergency non-trauma laparotomy. We grouped them based on transfusion exposure status. Transfusion exposure and possible confounders were recorded on entry, while the presence or absence of SSIs and mortality were obtained over 30 days of follow-up. The data were analyzed using Epi Info version 7 and Stata version 14. P-values <0.05 indicated statistical significance.

Results

All 160 participants recruited, 28 (17.5%) transfusion-exposed and 132 (82.5%) non-exposed, were included in the final analysis. Transfusion exposure (relative risk = 8.16; 95% confidence interval (CI) = 2.73-24.37; p < 0.001) was an independent risk factor for SSI after multivariate logistic regression analysis adjusted for confounders. Inverse probability weighting with regression adjustment (IPWRA) revealed that transfusion exposure significantly increased the incidence of SSI by 36.2% (95% CI = 14.2%-58.2%; p = 0.001). Furthermore, transfusion exposure (hazard ratio (HR) = 3.62; 95% CI = 1.28-10.27; p = 0.015) and age ≥60 years (HR = 5.97; 95% CI = 1.98-18.01; p = 0.002) were independent risk factors for 30-day mortality after multivariate Cox regression analysis adjusted for confounders. IPWRA revealed that transfusion exposure significantly increased the incidence of mortality by 17.6% (95% CI = 1.4%-33.8%; p = 0.033).

Conclusions

This study suggests an independent association between perioperative blood transfusion and the occurrence of SSIs and mortality among patients undergoing emergency non-trauma laparotomy. A larger multicenter prospective cohort study considering more confounders and the use of established restrictive transfusion protocols is recommended.

## Introduction

Perioperative blood transfusion is often required for the management of anemia in patients undergoing laparotomy. It improves oxygen-carrying capacity and is life-saving, especially in cases of ongoing trauma-related or intravascular coagulopathy-related hemorrhage. However, blood transfusion is associated with several complications, including postoperative infection due to transfusion-related immunomodulation (TRIM) and infection (bacterial/viral) transmission, among others [1,2]. This is illustrated by numerous retrospective cohort studies in high-income countries that have identified perioperative allogeneic blood transfusion as a predictor of postoperative infection and mortality in patient populations. These include patient populations such as major emergency abdominal surgery (unadjusted 30-day serious complication rate or Clavien-Dindo score ≥3a: 60.1% vs. 28.1%, p < 0.001; adjusted 30-day mortality following red blood cell/fresh frozen plasma/platelet transfusion: hazard ratio (HR) = 4.10; 95% confidence interval (CI) = 2.50-6.80; p < 0.001) [3]; colorectal cancer surgery (mortality: 41.33% vs. 7.64%; HR = 2.75; 95% CI = 1.05-7.21) [4]; poor survival (HR = 3.18; 95% CI = 2.08-4.84) [5], and Crohn’s disease (surgical site infection (SSI) rate: 28.3% vs. 14.4%; odds ratio (OR) = 2.20; 95% CI = 1.80-2.70; p < 0.001) [6].

In Uganda, SSI (20.0%) and mortality within 30 days (15.6%) are among the most common post-laparotomy complications [7], and, in this setting, emergency laparotomy constitutes 67.1% of laparotomies [8]. Generally, emergency laparotomies are frequently conducted with little patient preparation, thus often resulting in more postoperative complications than elective laparotomies (33.7% vs. 14.2%, p < 0.05) [9]. At the Mulago National Referral Hospital (MNRH), there is no consistently applied protocol for the transfusion of patients who undergo emergency non-trauma laparotomy as patients can be transfused for several reasons, including a liberal hemoglobin trigger level of <10 g/dL, severe illness on general examination, presence of signs of sepsis, and a low level of consciousness and shock. Consequently, approximately one in every five patients undergoing emergency non-trauma laparotomy in this hospital receives a blood transfusion (unpublished pilot study data, Egbe FE, 2022). However, no study has explored the association between perioperative blood transfusion and postoperative complications in this setting.

This study aims to assess the effect of perioperative blood transfusion on the 30-day risk of SSI and mortality among patients undergoing emergency laparotomy for gut perforation and bowel obstruction at MNRH, a large urban tertiary hospital in Uganda. Exploring this association in this resource-limited setting, where blood supply is limited compared to demand, may inform blood transfusion policies to reduce unnecessary blood transfusions and improve the utilization of scarce blood transfusion resources in Uganda and similar resource-limited settings.

A previous version of this article was presented as a meeting abstract at the Association of Surgeons of Uganda Annual Scientific Conference on March 28, 2024.

## Materials and methods

Study design and setting

This prospective, single-center, cohort study was conducted at the MNRH over 26 weeks between February 17, 2023, and August 17, 2023. This hospital is the teaching hospital of the College of Health Sciences, Makerere University, and Uganda’s main tertiary hospital. It serves several regional referral hospitals and other lower-level referral health facilities in Uganda and neighboring countries, including South Sudan and the Democratic Republic of the Congo. This institution’s high influx of patients is due to its free services and the concentration of several super-specialized units. This referral hospital’s Accident & Emergency Department includes a holding area, an operating theater, and a resuscitation unit. On average, 28 patients undergo emergency non-trauma laparotomy monthly in the operating theater of this unit, with approximately one out of every five patients receiving a blood transfusion (unpublished pilot study data, Egbe FE, 2022). Postoperative management of these patients is performed in the general surgery wards (endocrine, hepatobiliary, and colorectal surgery wards).

Study population and data collection

The study was approved by the Makerere University School of Medicine Research Ethics Committee (Mak-SOMREC-2022-494; 09-02-2023) and the MNRH Research Ethics Committee (MHREC 2432; 17-02-2023). Before inclusion in the study, written informed consent was obtained from each participant.

Participants in this study included all consecutively recruited adult patients (≥18 years) admitted to the general surgery wards of MNRH following emergency non-trauma laparotomy, who stayed beyond 48 hours after surgery, and who provided their written consent for inclusion in the study. The participants were recruited into either a transfusion-exposed or a non-exposed group. The transfusion-exposed group included participants who were transfused with whole blood or packed cells within 48 hours before surgery (preoperative period), during surgery (intraoperative period), or within 48 hours after surgery (postoperative period). The non-exposed group included participants who had not been transfused with any of the above blood products within the abovementioned period. However, all participants transfused within 30 days before the preoperative period were excluded from the study. The rationale for the choice of 48 hours is that from observation, most patients routinely undergo emergency laparotomy within 48 hours of presentation and those requiring transfusion receive it within 48 hours before and after surgery.

Independent/predictor variables, including age, measured in completed years, sex, history of smoking, and comorbidities, including the presence or absence of diabetes mellitus, immunodeficiency/human immunodeficiency virus seropositivity, and malignancy, were obtained by interviewing the participants and/or reviewing any formal test results or current drug treatment. Age was recorded as young-to-middle-age adults (18-59 years) and elderly adults (≥60 years). The participants’ body mass indices (BMIs) were obtained after their weights were measured with a weighing scale and their heights were measured with a measuring tape. BMI was recorded as the presence (BMI ≥30 kg/m^2^) or absence (BMI <30 kg/m^2^) of obesity and the presence (BMI <18.5 kg/m^2^) or absence (BMI ≥18.5 kg/m^2^) of being underweight. Perioperative blood transfusion exposure or non-exposure was obtained from participants’ records. Preoperative hemoglobin was obtained from participants’ preoperative complete blood count (CBC) results. This parameter was recorded as preoperative anemia (hemoglobin <10 g/dL) versus no preoperative anemia (hemoglobin ≥10 g/dL). Intraoperative findings were obtained from participants’ postoperative notes and included the following: American Society of Anesthesiologists (ASA) score (recoded as high at III/IV/V vs. low at I/II); cadre of the primary surgeon (a resident/trainee vs. a registrar/specialist); intraoperative diagnosis (classified as gut perforation vs. bowel obstruction); and duration of surgery (recoded as normal if ≤120 minutes vs. prolonged if >120 minutes). The latter cut-off for the duration of surgery is based on a recent study by Kaushal-Deep et al. (2019) in India that reported acceptable limits of mortality (≈5%) in pediatric emergency surgery in a resource-limited setting if the duration of surgery did not exceed 123.5 minutes [10]. Gut perforation included gastroduodenal, ileal, appendicular, gallbladder, and colonic perforations; bowel obstruction included obstructed/strangulated internal/abdominal wall hernia, postoperative adhesion, intussusception, volvulus, and colorectal tumor. The type of surgical procedure was recorded as either a procedure with a high risk for bowel leakage (if there was bowel perforation or resection and intraperitoneal bowel repair and included gut perforation repair, resection/anastomosis, abscess drainage, appendectomy, and bile drainage) or a procedure with low risk for bowel leakage (if there was no bowel perforation or peritoneal exposure to bowel content, or if there was external deviation of bowel content and included release of adhesions, hernia reduction/repair, volvulus de-rotation, intussusception reduction, and resection/stoma).

Dependent/outcome variables, including SSI and/or mortality, were obtained upon interval follow-up and clinical review of participants in both groups on postoperative days 3, 7, 14, 21, and 30. SSI was defined as pain/swelling/warmth/erythema and pus discharge involving the skin/subcutaneous tissue (superficial), pus discharge from the fascial/muscle layers around the surgical wound site with or without wound gaping/dehiscence (deep), and/or pus in the peritoneal cavity around organs and spaces explored during surgery (organ/space) occurring within 30 days of emergency non-trauma laparotomy. Mortality was defined as the death of a patient within 30 days of emergency non-trauma laparotomy. Participants discharged from the hospital before postoperative day 30 and unable to return to the hospital were followed up via telephone and video call interviews.

Sample size estimation and statistical analysis

The Fleiss formula for independent cohort studies estimated the sample size assuming a 5% level of significance and 80% power. According to the retrospective cohort study conducted on patients receiving transfusion in major emergency laparotomy by Schack et al. [3], 60.1% of the transfused patients experienced severe surgical complications while 28.1% of the non-transfused patients experienced complications (p < 0.001). In addition, in a recent pilot study based on records of the Accident & Emergency Department operating theater and blood bank of MNRH, 17% of patients who underwent emergency non-trauma laparotomy were transfused, and 83% were not. This implies that the ratio of unexposed to transfusion-exposed patients, m is 83/17 = 4.89 (unpublished pilot study data, Egbe FE, 2022). Therefore, a sample size of 160 participants (exposed = 28; non-exposed = 132) was obtained following a 10% adjustment for loss to follow-up.

All statistical analyses were performed using Epi Info version 7.2.5.0 (Centers for Disease Control and Prevention) and Stata/MP version 14.0 (StataCorp LP, College Station, TX, USA). Categorical data are presented as absolute numbers and percentages, n (%), and continuous variables with skewed distributions are expressed as medians ± interquartile range. Bivariate analyses involving categorical independent variables stratified by the transfusion group were performed using two-sided Fisher’s exact tests, while analyses involving continuous independent variables stratified by the transfusion group were performed using the Mann-Whitney U test. A two-sided p-value <0.05 was considered to indicate statistical significance. The data distribution is presented using frequency tables.

To assess the association between perioperative blood transfusion and the complications (30-day risk of SSI and mortality), univariate logistic regression analysis was used to determine the associations between independent variables and 30-day SSI. In addition, univariate Cox regression analysis was used to determine the associations between independent variables and 30-day mortality. All independent variables with p-values <0.1 were considered for the multivariate model. A test for multicollinearity was performed using variance inflation factor (VIF) analysis, but no variable was dropped from the multivariate model. Multivariate logistic regression analysis was used to assess the association between perioperative blood transfusion and the 30-day risk of SSI adjusted for confounders. These associations are reported as relative risks (RRs) with 95% CIs and p values. Furthermore, multivariate Cox regression analysis was used to assess the association between perioperative blood transfusion and 30-day mortality adjusted for confounders. These associations are reported as HRs with 95% CIs and p values. To account for any differences in independent variables between the transfusion-exposed and the non-exposed patients, we performed inverse probability weighting with regression adjustment (IPWRA). The treatment model included preoperative anemia, high ASA score, female sex, and registrar as the cadre of the primary surgeon which were associated with transfusion; the outcome model for SSI included preoperative anemia, young-to-middle-age adults, female sex, and gut perforation diagnosis which were associated with SSI; and the outcome model for mortality included elderly age group, immunodeficiency, and high ASA score which were associated with mortality. The average treatment effect on the treated (ATET) is reported as percentages with 95% CIs and p-values. Statistical significance was defined by a two-sided p-value <0.05.

## Results

**Figure 1:**
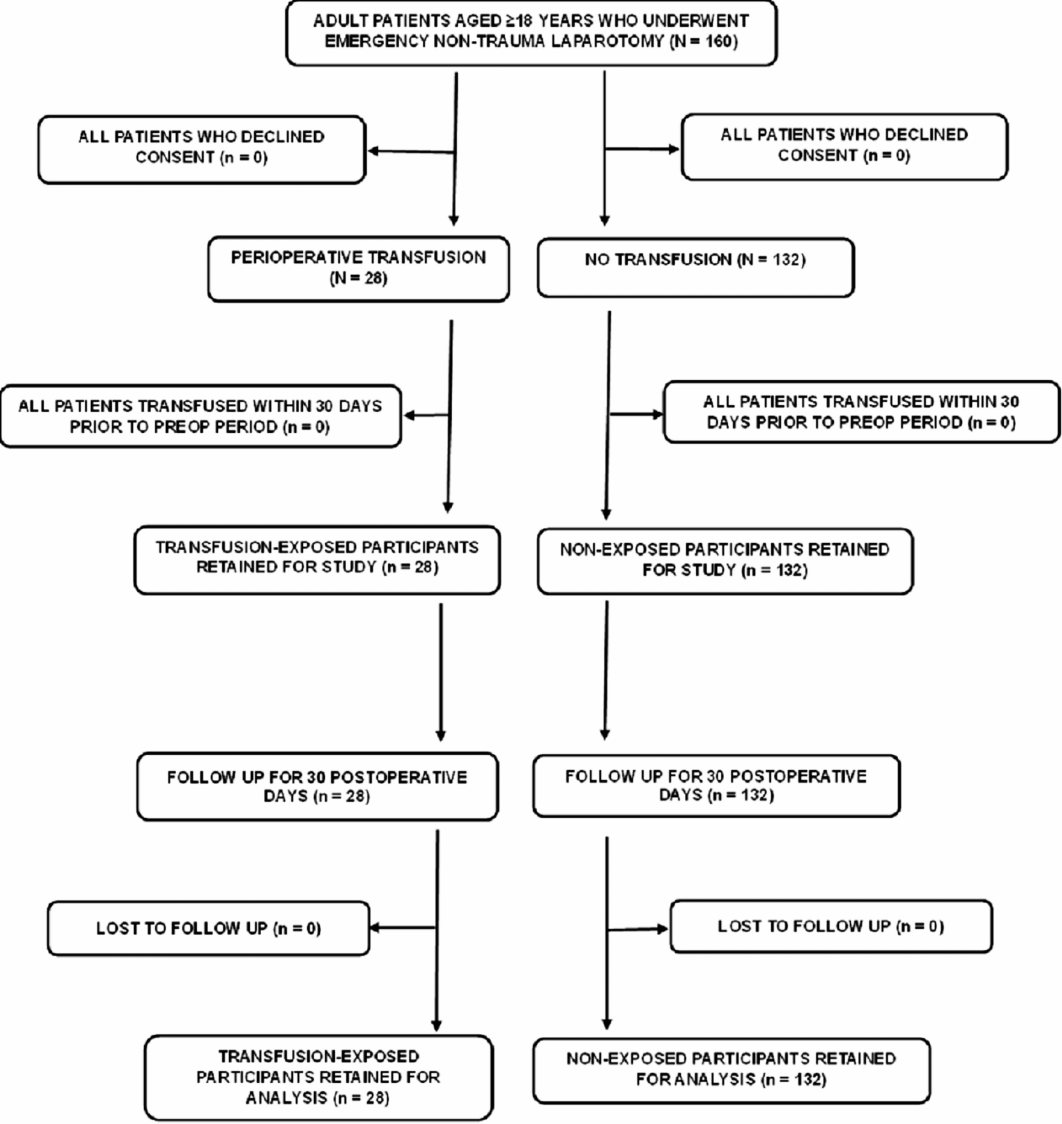
Flowchart showing participants’ recruitment into the study. Based on the inclusion criteria, 160 adult participants were recruited into the study; no patients were excluded. All participants were included in the final analysis.

Over the study period, 160 adult participants undergoing emergency non-trauma laparotomy were recruited: 28 (17.5%) were transfusion-exposed, and 132 (82.5%) were non-exposed. All participants completed the 30-day follow-up evaluation and were included in the final analysis, as shown in Figure 1.

Of those who were transfused, 28.6% (n = 8) of the participants were transfused for the first time within 48 hours before surgery, 53.6% (n = 15) were transfused during surgery, and 17.8% (n=5) received their transfusion within 48 hours after surgery. Table 1 shows that transfusion-exposed patients were significantly more likely to have a high ASA score (26.4%, n = 14 vs. 13.1%, n = 14; p = 0.047), be operated by a surgical team headed by a registrar (40.0%, n = 6 vs. 15.2%, n = 22; p = 0.027), have preoperative anemia (76.9%, n = 10 vs. 12.2%, n = 18; p < 0.001), and be of the female sex (27.7%, n = 13 vs 13.3%, n = 15; p = 0.039). However, the rest of the covariates were comparable between the groups (Table 1).

**Table 1:** Characteristics of the study population stratified by transfusion. ASA = American Society of Anesthesiologists. This score evaluates overall preoperative wellness and fitness for surgery requiring anesthesia. BMI = Body mass index. It indirectly measures body fatness and is used to screen for weight categories. **: p < 0.05 indicates statistical significance. ^a^: Mann-Whitney U test was used to analyze these variables. The rest of the variables were analyzed using Fisher’s exact test.

Variable	Total, n = 160	Transfusion-exposed, n = 28 (17.5%)	Non-exposed, n = 132 (82.5%)	P-value
^a^Age (years), median (Q1, Q3)	37 (23.0, 50.0)	36.0 (21.0, 48.0)	37 (24.0, 51.0)	0.639
Sex				
Female	47	13 (27.7)	34 (72.3)	0.039**
Male	113	15 (13.3)	98 (86.7)	
Preoperative anemia				
Yes	13	10 (76.9)	3 (23.1)	<0.001**
No	147	18 (12.2)	129 (87.8)	
Cadre of primary surgeon				
Registrar	15	6 (40.0)	9 (60.0)	0.027**
Resident	145	22 (15.2)	123 (84.8)	
ASA score				
High (III, IV, V)	53	14 (26.4)	39 (73.6)	0.047**
Low (I, II)	107	14 (13.1)	93 (86.9)	
Immunodeficiency				
Yes	14	5 (35.7)	9 (64.3)	0.073
No	146	23 (15.8)	123 (84.2)	
Malignancy				
Yes	18	5 (27.8)	13 (72.2)	0.319
No	142	23 (16.2)	119 (83.8)	
^a^Surgery duration (minutes), median (Q1, Q3)	110.0 (80.0, 150.0)	102.5 (85.0, 177.5)	110 (76.5, 150.0)	0.449
Type of surgical procedure				
High risk for bowel leak	111	18 (16.2)	93 (83.8)	0.508
Low risk for bowel leak	49	10 (20.4)	39 (79.6)	
Diabetes mellitus				
Yes	5	0 (0.0)	5 (100.0)	0.588
No	155	28 (18.1)	127 (81.9)	
^a^BMI (kg/m^2^), median (Q1, Q3)	20.6 (18.6, 22.3)	20.2 (18.0, 22.6)	20.6 (18.8, 22.3)	0.689
Smoking				
Yes	18	3 (16.7)	15 (83.3)	1.000
No	142	25 (17.6)	117 (82.4)	
Diagnosis				
Bowel obstruction	74	13 (17.6)	61 (82.4)	1.000
Gut perforation	86	15 (17.4)	71 (82.6)	

Association between perioperative blood transfusion and 30-day risk of surgical site infection in the unweighted cohort

In this study, the overall incidence of SSI was 34.4% (n = 55). The incidence of SSI in the transfusion-exposed group was 71.4% (n = 20) and in the non-exposed group was 26.5% (n = 35). Table 2 shows a univariate logistic regression analysis of the factors associated with 30-day SSI. Based on the p < 0.1 significance level, perioperative blood transfusion exposure (71.4%, n = 20 vs. 26.5%, n = 35; p < 0.001), preoperative anemia (61.5%, n = 8 vs. 32.0%, n = 47; p = 0.040), young-to-middle-age adults (37.6%, n = 50 vs. 18.5%, n = 5; p = 0.064), gut perforation diagnosis (40.7%, n = 35 vs. 27.0%, n = 20; p = 0.071), and female sex (40.7%, n = 21 vs. 30.1%, n = 34; p = 0.079) were considered for multivariate analysis (Table 2).

**Table 2:** Thirty-day risk of surgical site infection based on univariate logistic regression analysis. ASA = American Society of Anesthesiologists. This score evaluates overall preoperative wellness and fitness for surgery requiring anesthesia. BMI = Body mass index. It indirectly measures body fatness and is used to screen for weight categories. *: p < 0.1 indicates statistical significance following univariate logistic regression analysis. CI = Confidence interval of the relative risk of the associated factors of surgical site infection.

Variable	Total, n = 160	Surgical site infection (%)		Relative risk (95% CI)	P-value
		Yes	No		
Perioperative transfusion exposure					
No	132	35 (26.5)	97 (73.5)	1	<0.001 *
Yes	28	20 (71.4)	8 (28.6)	6.93 (2.80–17.12)	
Preoperative anemia					
No	147	47 (32.0)	100 (68.0)	1	0.040 *
Yes	13	8 (61.5)	5 (38.5)	3.40 (1.06–10.97)	
Age					
Elderly adults (≥60 years)	27	5 (18.5)	22 (81.5)	1	0.064 *
Young-to-middle-age adults (18–59 years)	133	50 (37.6)	83 (62.4)	2.65 (0.94–7.44)	
Diagnosis					
Bowel obstruction	74	20 (27.0)	54 (73.0)	1	0.071 *
Gut perforation	86	35 (40.7)	51 (59.3)	1.85 (0.95–3.62)	
Sex					
Male	113	34 (30.1)	79 (69.9)	1	0.079*
Female	47	21 (44.7)	26 (55.3)	1.88 (0.93–3.79)	
Cadre of the primary surgeon					
Resident	145	47 (32.4)	98 (67.6)	1	0.112
Registrar	15	8 (53.3)	7 (46.7)	2.38 (0.82–6.96)	
Type of surgical procedure					
Low risk for bowel leak	49	13 (26.5)	36 (73.5)	1	0.167
High risk for bowel leak	111	42 (37.8)	69 (62.2)	1.69 (0.80–3.54)	
BMI = Underweight					
No	123	40 (32.5)	83 (67.5)	1	0.369
Yes	37	15 (40.5)	22 (59.5)	1.41 (0.66–3.02)	
Immunodeficiency					
No	146	49 (33.6)	97 (66.4)	1	0.486
Yes	14	6 (42.9)	8 (57.1)	1.49 (0.49–4.52)	
BMI = Obesity					
No	156	53 (34.0)	103 (66.0)	1	0.512
Yes	4	2 (50.0)	2 (50.0)	1.94 (0.27–14.19)	
Malignancy					
No	142	50 (35.2)	92 (64.8)	1	0.533
Yes	18	5 (27.8)	13 (72.2)	0.71 (0.24–2.10)	
ASA score					
Low (I, II)	107	36 (33.6)	71 (66.4)	1	0.782
High (III, IV, V)	53	19 (35.9)	34 (64.1)	1.10 (0.55–2.20)	
Diabetes mellitus					
No	155	53 (34.2)	102 (65.8)	1	0.788
Yes	5	2 (40.0)	3 (60.0)	1.28 (0.21–7.92)	
Smoking					
No	142	49 (34.5)	93 (65.5)	1	0.921
Yes	18	6 (33.3)	12 (66.7)	0.95 (0.34–2.68)	
Surgery duration					
Normal	99	34 (34.3)	65 (65.7)	1	0.991
Prolonged	61	21 (34.4)	40 (65.6)	1.00 (0.51–1.97)	

Following the VIF analysis for multicollinearity, no variable was dropped from the multivariate regression model because none of them was collinear. Multivariate logistic regression analysis (method: forced entry), which adjusted for confounders, revealed that transfusion exposure was an independent risk factor for 30-day SSI among participants undergoing emergency non-trauma laparotomy at MNRH, Kampala, Uganda. Perioperative blood transfusion exposure resulted in an 8.16-fold greater risk of SSI within 30 days of emergency non-trauma laparotomy than non-exposure (RR = 8.16; 95% CI = 2.73-24.37; p < 0.001) (Table 3).

**Table 3:** Thirty-day risk of surgical site infection based on multivariate logistic regression analysis. **: p < 0.05 indicates statistical significance following multivariate logistic regression analysis. CI = Confidence interval of the relative risk of the risk factor of surgical site infection.

Variable	Relative risk (95% CI)	P-value
Perioperative blood transfusion exposure		
No	1	<0.001**
Yes	8.16 (2.73–24.37)	
Diagnosis		
Bowel obstruction	1	0.104
Gut perforation	1.92 (0.88–4.19)	
Age		
Elderly adults (≥ 60 years)	1	0.106
Young-to-middle age adults (18 – 59 years)	2.60 (0.82–8.26)	
Sex		
Male	1	0.162
Female	1.77 (0.80–3.94)	
Preoperative anemia		
No	1	0.618
Yes	0.68 (0.15–3.14)	

Effect of perioperative blood transfusion on the 30-day risk of surgical site infection in the inverse probability-weighted cohort

To account for any differences in independent variables between the transfusion-exposed and the non-exposed patients (confounding by indication), we performed the doubly robust IPWRA. This method uses a treatment regression model to estimate propensity scores (patient’s likelihood of being exposed to transfusion) and inverse probability weights for each participant adjusted for variables associated with the treatment. It then uses an outcome regression model that fits variables associated with the outcome and the inverse probability weights to estimate the strength of association with the outcome. In the current analysis, preoperative anemia, ASA score, female sex, and the cadre of the primary surgeon were included in the treatment model as they were associated with the treatment (transfusion). Conversely, variables associated with the outcome (SSI), such as young-to-middle-age adult, gut perforation diagnosis, female sex, and preoperative anemia, were included in the outcome model.

We found that the average incidence of SSI was 36.2% (95% CI = 14.2%-58.2%; p = 0.001), significantly greater among participants who were transfused than the average incidence of 35.2% that would have occurred if this transfused population was not transfused (Table 4).

**Table 4:** Estimate of the average effect of transfusion (surgical site infection) on the transfused group based on inverse probability weighting with regression adjustment. Outcome model: logit; treatment model: probit; standard error: robust. **: p < 0.05 denotes statistical significance following inverse probability weighting with regression adjustment analysis. CI = Confidence interval for the average treatment effect on the treated.

Surgical site infection	Coefficient (95% CI)	P-value
Average treatment effect on treated (transfusion exposed vs. non-exposed)	0.362 (0.142–0.582)	0.001**
Potential outcome mean (non-exposed)	0.352 (0.186–0.519)	<0.001**

Association between perioperative blood transfusion and 30-day mortality risk in the unweighted cohort

In this study, the overall incidence of 30-day mortality was 9.4% (n = 15). The incidence of 30-day mortality in the transfusion-exposed group was 25.0% (n = 7) and the 30-day mortality in the non-exposed group was 6.1% (n = 8). Table 5 shows a univariate Cox regression analysis of the factors associated with 30-day mortality. Based on the p < 0.1 significance level, perioperative blood transfusion exposure (25.0%, n = 7 vs. 6.1%, n = 8; p = 0.003), old age ≥60 years (28.6%, n = 8 vs. 5.3%, n = 7; p < 0.001), high ASA score (18.9%, n = 10 vs. 4.7%, n = 5; p = 0.009), and immunodeficiency (28.6%, n = 4 vs. 7.5%, n = 11; p = 0.013) were considered for multivariate analysis (Table 5).

**Table 5:** Thirty-day risk of mortality based on univariate Cox regression analysis. ASA = American Society of Anesthesiologists. This score evaluates overall preoperative wellness and fitness for surgery requiring anesthesia. BMI = Body mass index. It indirectly measures body fatness and is used to screen for weight categories. CI = Confidence interval of the hazard ratio of the associated factors of mortality. *: p < 0.1 indicates statistical significance following univariate Cox regression analysis.

Variable	Total, n = 160	Mortality (%)		Hazard ratio (95% CI)	P-value
		Yes	No		
Perioperative transfusion exposure					
No	132	8 (6.1)	124 (93.9)	1	0.003*
Yes	28	7 (25.0)	21 (75.0)	4.55 (1.65–12.56)	
Age					
Young-to-middle age adults (18–59 years)	132	7 (5.3)	125 (94.7)	1	<0.001*
Elderly adults (≥60 years)	28	8 (28.6)	20 (71.4)	6.19 (2.24–17.09)	
ASA score					
Low (I, II)	107	5 (4.7)	102 (95.3)	1	0.009 *
High (III, IV, V)	53	10 (18.9)	43 (81.1)	4.19 (1.43–12.26)	
Immunodeficiency					
No	146	11 (7.5)	135 (92.5)	1	0.013 *
Yes	14	4 (28.6)	10 (71.4)	4.28 (1.36–13.45)	
Diagnosis					
Gut perforation	86	5 (5.8)	81 (94.2)	1	0.106
Bowel obstruction	74	10 (13.5)	64 (86.5)	2.42 (0.83–7.09)	
Type of surgical procedure					
Low risk for bowel leak	49	7 (14.3)	42 (85.7)	1	0.169
High risk for bowel leak	111	8 (7.2)	103 (92.8)	0.49 (0.18–1.35)	
Surgical site infection					
No	105	8 (7.6)	97 (92.4)	1	0.305
Yes	55	7 (12.7)	48 (87.3)	1.70 (0.62–4.69)	
BMI = Underweight					
No	123	13 (10.6)	110 (89.4)	1	0.356
Yes	37	2 (5.4)	35 (94.6)	0.50 (0.11–2.20)	
Surgery duration					
Normal	61	4 (6.6)	57 (93.4)	1	0.366
Prolonged	99	11 (11.1)	88 (88.9)	1.70 (0.54–5.32)	
Diabetes mellitus					
No	155	14 (9.0)	141 (91.0)	1	0.425
Yes	5	1 (20.0)	4 (80.0)	2.28 (0.30–17.37)	
Preoperative anemia					
No	147	13 (8.8)	134 (91.2)	1	0.427
Yes	13	2 (15.4)	11 (84.6)	1.83 (0.41–8.10)	
Sex					
Male	113	10 (8.9)	103 (91.1)	1	0.718
Female	47	5 (10.6)	42 (89.4)	1.22 (0.42–3.57)	
Malignancy					
No	142	13 (9.2)	129 (90.8)	1	0.758
Yes	18	2 (11.1)	16 (88.9)	1.26 (0.29–5.60)	
Smoking					
No	142	13 (9.2)	129 (90.8)	1	0.834
Yes	18	2 (11.1)	16 (88.9)	1.17 (0.27–5.20)	
Cadre of the primary surgeon					
Registrar	16	0 (0.0)	16 (100.0)	1	0.997
Resident	144	15 (10.4)	129 (89.6)	>10,000 (0.00–>10,000)	
BMI = Obesity					
No	156	15 (9.6)	141 (90.4)	1	0.999
Yes	4	0 (0.0)	4 (100.0)	0.00 (0.00–>10000)	

Following the VIF analysis for multicollinearity, no variable was dropped from the multivariate Cox regression model because none of them was collinear. Multivariate Cox regression analysis (method: forced entry), which adjusted for confounders, identified transfusion exposure and old age as independent risk factors for 30-day mortality among participants undergoing emergency non-trauma laparotomy at MNRH, Kampala, Uganda. Patients exposed to perioperative blood transfusion had a 3.62-fold greater mortality risk within 30 days of emergency non-trauma laparotomy than patients who were not exposed (HR = 3.62; 95% CI = 1.28-10.27; p = 0.015). Furthermore, adults aged 60 years and above had a 5.97-fold greater risk of mortality than adults aged less than 60 years (HR = 5.97; 95% CI = 1.98-18.01; p = 0.002) (Table 6).

**Table 6:** Thirty-day risk of mortality based on multivariate Cox regression analysis. ASA = American Society of Anesthesiologists. This score evaluates overall preoperative wellness and fitness for surgery requiring anesthesia. CI = Confidence interval of the hazard ratio of the risk factors of mortality. **: p < 0.05 indicates statistical significance following multivariate Cox regression analysis.

Variable	Hazard ratio (95% CI)	P-value
Perioperative blood transfusion exposure		
No	1	0.015**
Yes	3.62 (1.28–10.27)	
Age		
Young-to-middle-age adults (18–59 years)	1	0.002**
Elderly adults (≥60 years)	5.97 (1.98–18.01)	
Immunodeficiency		
No	1	0.079
Yes	2.83 (0.89–9.01)	
ASA score		
Low (I, II)	1	0.284
High (III, IV, V)	1.95 (0.57–6.66)	

Effect of perioperative blood transfusion on the 30-day mortality risk in the inverse probability-weighted cohort

We also conducted the doubly robust IPWRA to account for any differences in independent variables between the transfusion-exposed and the non-exposed patients (confounding by indication). Preoperative anemia, high ASA score, female sex, and registrar as the cadre of the primary surgeon were included in the treatment model as they were associated with the treatment (transfusion). The elderly age group, high ASA score, and immunodeficiency, which were associated with the outcome (mortality), were included in the outcome model.

We found that the average incidence of mortality was 17.6% (95% CI = 1.4%-33.8%; p = 0.033), significantly greater among participants who were transfused than the average incidence of 7.4% that would have occurred if this transfused population was not transfused (Table 7).

**Table 7:** Estimate of the average effect of transfusion (30-day mortality) on the transfused group based on inverse probability weighting with regression adjustment. Outcome model: logit; treatment model: probit; standard error: robust. **: p < 0.05 denotes statistical significance following inverse probability weighting with regression adjustment analysis. CI = Confidence interval for the average treatment effect on the treated.

Mortality	Coefficient (95% CI)	P-value
Average treatment effect on treated (transfusion exposed vs. non-exposed)	0.176 (0.014–0.338)	0.033**
Potential outcome mean (non-exposed)	0.074 (-0.011–0.159)	0.090

## Discussion

This prospective cohort study which aimed to investigate the effect of blood transfusion on postoperative complications following emergency non-trauma laparotomy showed an independent association between perioperative blood transfusions and SSI. In addition, the study revealed an independent association between perioperative blood transfusion and age ≥60 years and 30-day mortality. Following IPWRA for variables noted to be associated with perioperative transfusion, SSI, and mortality to account for confounding by indication, the above independent association between transfusion and SSI and mortality remained the same.

This susceptibility of transfusion-exposed patients to surgical site infection was postulated to result from the immune modulation induced by transfusion and the current study lends evidence to same. This TRIM phenomenon was first described over three decades ago in renal transplant recipients who demonstrated improved allograft survival after allogeneic blood transfusion. It was postulated that exposure to allogeneic blood transfusions induces clinically significant immunosuppression and allo-sensitization in recipients [11,12]. This eventually leads to postoperative bacterial infection and cancer recurrence while improving outcomes in renal allograft transplantation [13]. In addition, previous studies have shown that major emergency surgery, including laparotomy, contributes to immunomodulation by suppressing T helper (TH) cell function during the early postoperative period and reducing the secretion of interleukin (IL)-2, interferon-gamma, tumor necrosis factor-alpha (which stimulate TH1), and IL-4 (which stimulate TH2), thus increasing host susceptibility to septic complications [14]. Furthermore, perioperative allogeneic blood transfusion worsens stress-induced postsurgical immunosuppression by impairing natural killer cell activity [15]. SSI is the most common complication of laparotomy in Uganda [7], and this complication is generally associated with increased morbidity (pain, prolonged postoperative hospital stay, and readmission resulting in a negative economic impact on the patient and the health system) and mortality due to sepsis [16]. The finding of this study is consistent with several recently published findings from high-income countries suggesting that perioperative blood transfusion is an independent risk factor for SSI. These include retrospective cohort studies of various patient populations, viz, patients undergoing major emergency laparotomy (Schack et al., 2021) [3], colorectal cancer complications (Jiang et al., 2022) [4], colorectal cancer surgery (McSorley et al., 2020) [5], surgery for Crohn’s disease (Lan et al., 2018) [6], gastrectomy for gastric cancer (Xue et al., 2016) [17], pancreaticoduodenectomy (Dosch et al., 2019) [18] and in non-neonatal pediatric general and thoracic surgical patients, which revealed an association between blood transfusion and an increased risk of postoperative complications [19].

The association between transfusion exposure and mortality in this study could be attributed to the transfusion-exposed patients being inherently sicker than the non-exposed patients as shown by the significantly greater proportion of acutely sicker patients (ASA ≥3) who were transfused than less sick patients (ASA ≤2). Furthermore, in this study, a high ASA score (ASA ≥3) was associated with mortality among patients undergoing emergency non-trauma laparotomy. This finding is similar to that of a recent retrospective cohort study in Ethiopia that reported a significant association between the ASA score and mortality among patients undergoing emergency laparotomy [20]. However, following risk adjustment analysis, while transfusion exposure was an independent risk factor for mortality in this study, being inherently sicker (ASA ≥3) was not an independent risk factor of mortality. These findings were consistent with recently published data that reported perioperative transfusion as an independent risk factor for mortality, such as the studies by Schack et al. (2022) involving patients undergoing major emergency laparotomy [3], Jiang et al. (2022) concerning colorectal cancer complications [4], and McSorley et al. (2020) involving patients undergoing colorectal cancer surgery [5]. In addition, Xue et al. (2016) asserted that perioperative transfusion was associated with poor survival in patients with preoperative hemoglobin (POHb) >100 g/L (p < 0.001), unlike in transfused patients with POHb between 70 and 100 g/L (p = 0.191) [17].

The current study also revealed that participants aged ≥60 years were more likely to die within 30 days of emergency non-trauma laparotomy than those aged <60 years. Elderly persons are known to have age-associated decreased physiological reserve in multiple systems. This results in decreased resilience to physiological insults such as laparotomy; hence, impairing recovery to pre-morbid functional levels [21]. Furthermore, stress-induced immunosuppression following surgery [14] may worsen these already frail elderly persons, resulting in worse outcomes than in younger adults. Our findings were consistent with data from various recent studies. Oumer et al. (2021) in Ethiopia found that the odds of dying after emergency laparotomy among patients aged ≥65 years were 10 times greater than those among patients aged <65 years [20], and Peponis et al. (2017) in a recent retrospective cohort study in the United States also revealed an association between age >60 years and mortality following emergency laparotomy [22].

There were a few limitations to this study. First, there was no transfusion protocol indicating a transfusion trigger or threshold. Consequently, some patients with anemia were not transfused, while others without anemia were transfused. Confounding by indication resulting from any differences in the independent variables between the transfusion-exposed and the non-exposed groups was reduced by performing the doubly robust IPWRA for covariates that were associated with transfusion, SSI, and mortality. Furthermore, this study was performed with a small sample size; hence, the reliability of our results was affected, as shown by the wide CIs for the measure of association. Further, variables including intraoperative blood loss, the volume of fluid used for peritoneal lavage, and preoperative sepsis which are important confounders were not considered in this study as these intraoperative findings and the parameters used to define preoperative sepsis were not consistently captured in patients’ postoperative notes used partly as the data source for this study. Finally, there could have been reporting bias given that patient-related variables such as diabetes, immunodeficiency, and malignancy were identified by prior diagnosis and as declared by the patient as no further diagnostic tests were performed.

A previous version of this article was posted to the Research Square preprint server on March 8, 2024 [23].

## Conclusions

Perioperative blood transfusion and postoperative complications including SSI and mortality are frequent occurrences following emergency non-trauma laparotomy. In this study, we demonstrated an independent association between perioperative blood transfusion and the occurrence of SSI and mortality, suggesting that the liberal perioperative blood transfusion currently practiced in this setting may harm outcomes of emergency non-trauma laparotomy. This study is Uganda’s first prospective cohort study to provide crucial contextual evidence of this phenomenon. This emphasizes the need for more substantial evidence of this phenomenon in a larger multicenter prospective cohort study. Such a study should consider more confounders, including estimated blood loss, the volume of fluid used for peritoneal lavage, preoperative sepsis, and other comorbidities, thereby addressing important limitations of the current study. Nevertheless, we recommend using established restrictive protocols for blood transfusion (transfusion trigger hemoglobin level of less than 8 g/dL) to reduce the rate of unnecessary blood transfusions and mitigate the effect of blood transfusion on outcomes of emergency non-trauma laparotomy.
